# Circadian Modulation of 8-Oxoguanine DNA Damage Repair

**DOI:** 10.1038/srep13752

**Published:** 2015-09-04

**Authors:** Nicola Manzella, Massimo Bracci, Elisabetta Strafella, Sara Staffolani, Veronica Ciarapica, Alfredo Copertaro, Venerando Rapisarda, Caterina Ledda, Monica Amati, Matteo Valentino, Marco Tomasetti, Richard G. Stevens, Lory Santarelli

**Affiliations:** 1 Occupational Medicine, Department of Clinical and Molecular Sciences, Polytechnic University of Marche, Via Tronto 10/A, 60126 Ancona, Italy; 2 Healthcare Workers Service, ASUR Area 2, Loreto Hospital, Via S. Francesco 1, 60025 Loreto, Italy; 3 Section of Occupational Medicine, Department of Internal Medicine and Systemic Diseases, University of Catania, Via Santa Sofia 78, 95123 Catania, Italy; 4 Hygiene and Public Health, Department “G.F. Ingrassia”, University of Catania, Via Santa Sofia 87, 95123 Catania, Italy; 5 Department of Community Medicine, University of Connecticut Health Center, 263 Farmington Avenue, Farmington, CT 06030-6325, USA

## Abstract

The DNA base excision repair pathway is the main system involved in the removal of oxidative damage to DNA such as 8-Oxoguanine (8-oxoG) primarily via the 8-Oxoguanine DNA glycosylase (OGG1). Our goal was to investigate whether the repair of 8-oxoG DNA damage follow a circadian rhythm. In a group of 15 healthy volunteers, we found a daily variation of *Ogg1* expression and activity with higher levels in the morning compared to the evening hours. Consistent with this, we also found lower levels of 8-oxoG in morning hours compared to those in the evening hours. Lymphocytes exposed to oxidative damage to DNA at 8:00 AM display lower accumulation of 8-oxoG than lymphocytes exposed at 8:00 PM. Furthermore, altered levels of *Ogg1* expression were also observed in a group of shift workers experiencing a deregulation of circadian clock genes compared to a control group. Moreover, BMAL1 knockdown fibroblasts with a deregulated molecular clock showed an abolishment of circadian variation of *Ogg1* expression and an increase of OGG1 activity. Our results suggest that the circadian modulation of 8-oxoG DNA damage repair, according to a variation of *Ogg1* expression, could render humans less susceptible to accumulate 8-oxoG DNA damage in the morning hours.

In order to ensure proper adaptation to constantly changing environmental conditions, living organisms have developed a molecular system called the “circadian clock” which is capable of generating 24-hour periodicities in various physiological and behavioral processes^1^,^2^.

Numerous epidemiological studies have revealed that alterations in sleep/wake or light/dark cycles such as those experienced by shift workers can result in perturbations in the functioning of the circadian clock associated with increased cancer risk^3^,^4^,^5^,^6^,^7^,^8^,^9^,^10^,^11^. At the molecular level, the control of the circadian clock is maintained by tightly controlled transcription/repression of both positive and negative factors involved in several interconnected feedback loops^1^,^12^.

Daily changes in the environment (cycles in light/darkness, feeding, rest/activity, and temperature fluctuations) inevitably expose the mammalian cells and tissues to periodic challenges, including oxidative insults from environmental toxins/pollutants and endogenously produced reactive metabolites such as products of respiration^13^. The ability to anticipate and withstand such cyclic insults is therefore essential for normal protective tissue functions. There is emerging evidence that circadian clocks regulate the cellular response to DNA-damage^14^,^15^.

Base excision repair (BER) is the main repair system for removing a wide range of damaged bases from DNA. Among them is oxidized guanine (8-oxoG) which has been shown to display mutagenic potential by inducing GC-TA transversion^16^,^17^. BER pathway is initiated by DNA glycosylases, primarily OGG1 which is a critical protein involved in damage recognition and a rate-limiting factor in excision repair^18^. A role of this enzyme in cancer prevention/progression has been documented^19^,^20^.

In the current study, we test the hypothesis that 8-oxoG DNA damage repair follows a circadian rhythm.

## Results

### Daily variation of cortisol and melatonin

**Figure 1:**
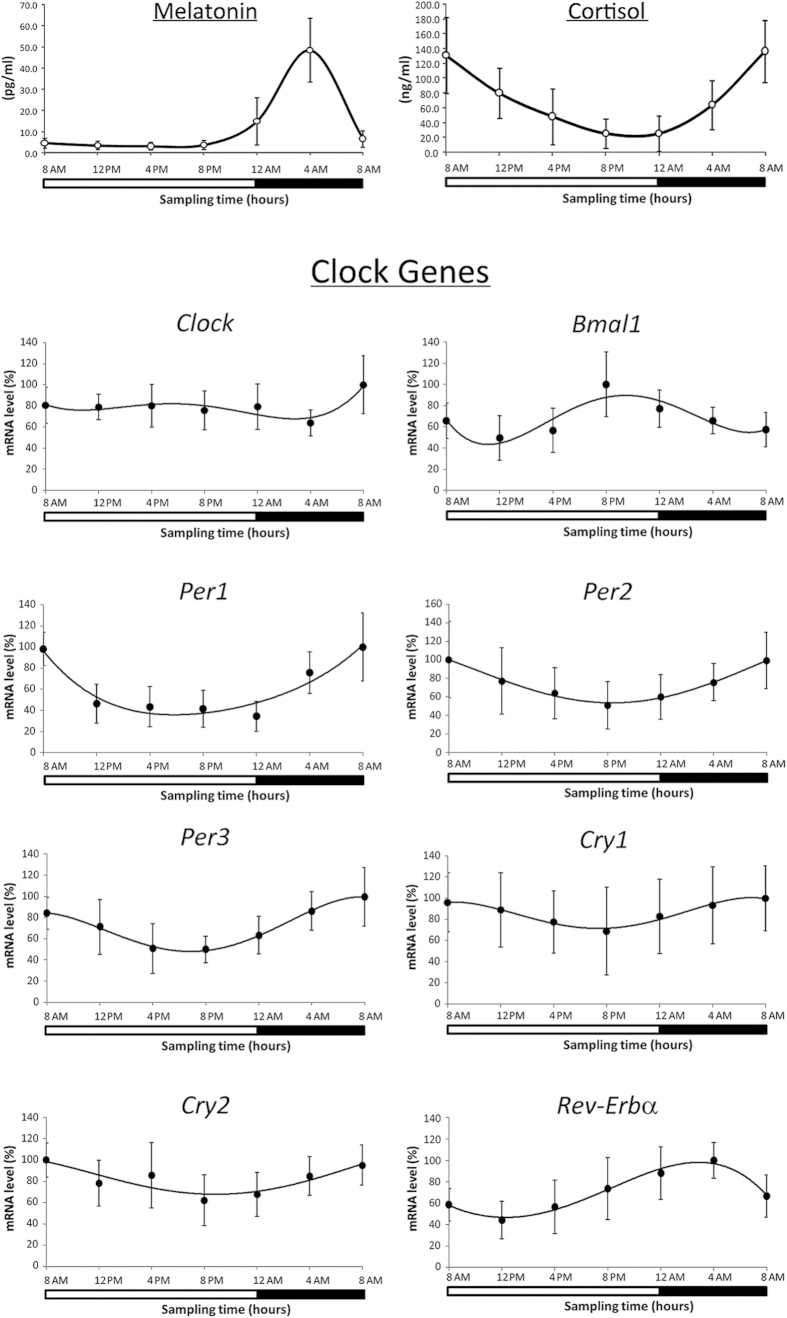
Melatonin and cortisol levels and expression profiles of peripheral clock genes in 15 subjects in blood samples collected every 4 h for a 24 h period. Data of melatonin and cortisol are expressed as mean ± SD. Data of mRNA levels (means ± SD) are showed as % relative levels compared to the max value (acrophase) set to 100%. The shaded region represents sleep time. A statistically significant circadian rhythm was validated for both melatonin and cortisol levels and for expression of all clock genes with the exception of *Clock* gene with ANOVA repeated measures and Cosinor analysis (p < 0.05).

The 24 h mean concentrations (±SD) were 11.9 ± 16.6 pg/ml for plasma melatonin and 78.1 ± 60.6 ng/ml for plasma cortisol in the group of 15 subjects. Mean plasma melatonin concentration was minimum at 4:00 PM (3.1 ± 1.6 pg/ml) and reached a maximum at 4:00 AM (48.4 ± 15.0 pg/ml). Mean plasma cortisol concentration decreased from 185.6 ± 54.0 ng/ml at 8:00 AM (peak) down to 21.8 ± 19.7 ng/ml at 8:00 PM (trough) (Fig. 1). A statistically significant circadian rhythm was validated for these hormonal levels with both ANOVA repeated measures and Cosinor analysis (p < 0.05). Cosinor analysis documented an acrophase at 3:50 AM for melatonin (p < 0.05) and 7:12 AM for cortisol (p < 0.05). Individual 24 h means varied 3.0-fold among subjects from 37.5 to 112.5 pg/ml for plasma melatonin concentration and 7.5-fold from 3.9 to 29.1 ng/ml for plasma cortisol concentration.

### Daily variation of clock gene expression

The temporal mRNA expression patterns of all clock genes examined in lymphocytes from 15 subjects showed a significant variation in the 24 h period with the exception of *Clock* gene (ANOVA repeated measures, p < 0.05) (Fig. 1).

*Bmal1* showed a significant difference in mRNA levels at 8:00 PM (peak) and at 12:00 PM (noon) (trough) (ANOVA measures repeated, post hoc LSD, p < 0.05). *Per* and *Cry* significantly decreased from peak at 8:00 AM to trough at 8:00 PM (ANOVA repeated measures, post hoc LSD, p < 0.05). *Rev-Erbα* gene showed a significant difference in mRNA levels at 4:00 AM (peak) and at 12:00 PM (trough) (ANOVA measures repeated, post hoc LSD, p < 0.05). The mRNA levels of all analyzed circadian genes (except *CLOCK*) showed a significant correlation with each other in the whole study group (data not shown). Furthermore, Pearson’s analysis demonstrated a significant correlation between cortisol levels and clock gene expression in all the subjects examined. Cosinor analysis confirmed results found by ANOVA analysis (data not shown).

### Daily variation of expression of selected genes of BER system

**Figure 2:**
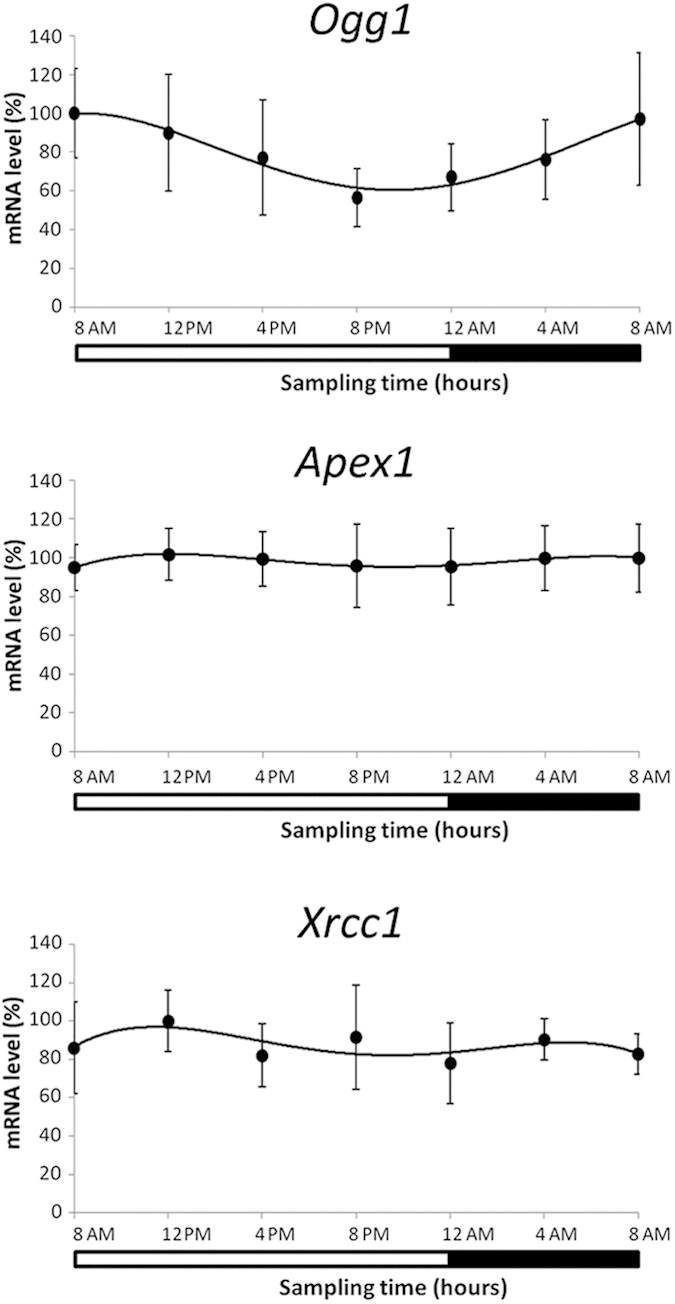
Expression profiles of selected genes of BER system in 15 subjects in lymphocytes collected every 4 h for a 24 h period. Data of mRNA levels (means ± SD) are showed as % relative levels compared to the max value (acrophase) set to 100%. The shaded region represents sleep time. A statistically significant circadian rhythm was validated for *Ogg1* mRNA with ANOVA repeated measures and Cosinor analysis (p < 0.05).

Among the selected genes of the BER system examined, only *Ogg1* exhibited a circadian time-dependent change in expression in the 24 h period with a higher level at 8:00 AM than that at 8:00 PM (ANOVA repeated measures, post hoc LSD, p < 0.05) (Fig. 2). *Ogg1* expression positively correlated with *Per* (*Per1* r = 0.76; *Per2* r = 0.95; *Per3* r = 0.75) and *Cry* (*Cry1* r = 0.86; *Cry2* r = 0.90) genes and negatively with *Bmal1* (r = −0.76) and *Rev-Erbα* (r = −0.84) (Pearson Correlation, p < 0.05). Moreover, Pearson’s analysis reported a positive correlation between the *Ogg1* expression and plasma cortisol concentration (r = 0.89, p < 0.05). Cosinor analysis confirmed results found by the ANOVA analysis and documented an acrophase at 7:45 AM for the circadian oscillation of *Ogg1* expression. No significant variation was found for *Apex1* and *Xrcc1* expression.

### Daily variation of OGG1 activity

**Figure 3:**
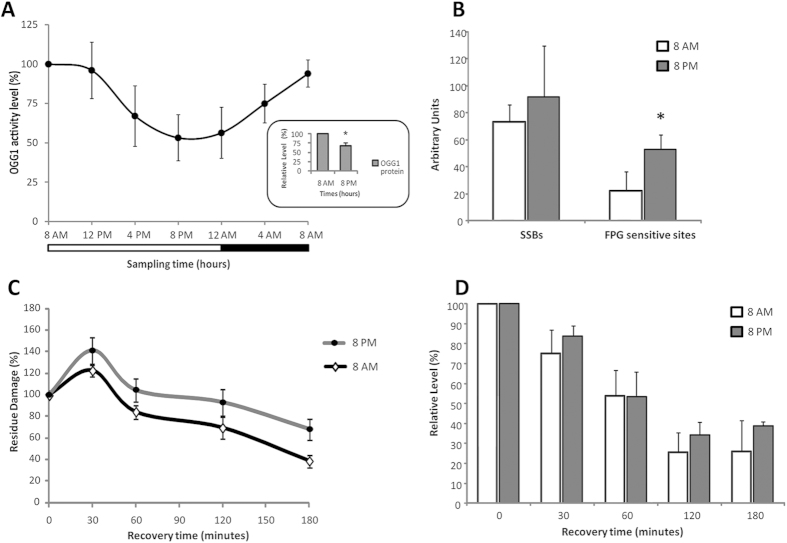
(**A**) Profile of OGG1 activity in 15 subjects in lymphocytes collected every 4 h for a 24 h period. Data (means ± SD) are showed as % relative levels compared to the max value (acrophase) set to 100%. The shaded region represents sleep time. A statistically significant circadian rhythm was validated for OGG1 activity with ANOVA repeated measures and Cosinor analysis (p < 0.05). The OGG1 protein levels at 8:00 AM and 8:00 PM are shown in the box of the figure. * = p < 0.05 8:00 PM vs 8:00 AM. (**B**) Levels of Single Strand Breaks (SSBs) and Formamidopyrimidine-DNA Glycosylases (FPG) sensitive sites at 8:00 AM and 8:00 PM. Data are expressed as the mean ± SD of Arbitrary Units. * = p < 0.05 8:00 PM vs 8:00 AM. (**C**) The kinetics of repair of FPG sensitive sites at 0, 30, 120 and 180 minutes after *ex vivo* oxidative treatment (Ro 198022) performed at 8:00 AM and 8:00 PM. The kinetics of DNA repair was calculated as a percentage of residue damage with respect to the damage at 0 minutes. (**D**) Levels of *Ogg1* expression at 0, 30, 120 and 180 minutes after *ex vivo* oxidative treatment (Ro 198022) performed at 8:00 AM and 8:00 PM. Data are expressed as % relative levels compared to the value at 0 minutes set to 100%.

Consistent with previous results of mRNA levels, OGG1 activity showed a significant variation in the 24 h period in each subject of this study with a peak at 8:00 AM and a trough at 8:00 PM (ANOVA repeated measures, post hoc LSD, p < 0.05) (Fig. 3A). A positive correlation (r = 0.960, p < 0.001) was found between OGG1 activity and *Ogg1* expression in the whole study group. An acrophase at 8:05 AM of OGG1 activity was reported by Cosinor analysis.

Furthermore, we analyzed OGG1 protein levels at peak (8:00 AM) and at trough (8:00 PM) of the circadian oscillation in OGG1 expression and activity. The densitometric analysis showed significantly lower levels of OGG1 protein at 8:00 PM than those at 8:00 AM in all the subjects examined (Student’s t-Test for paired samples, p < 0.05) (Fig. 3A).

### Oxidative damage to DNA at morning and evening times

Endogenous levels of Single Strand Breaks (SSBs) and Formamidopyrimidine-DNA Glycosylases (FPG) sensitive sites were evaluated at morning peak (8:00 AM) and evening trough (8:00 PM) of the circadian oscillation in OGG1 activity. No difference was found in SSBs levels between the two different time points (Student’s t-Test for paired samples, p > 0.05). The levels of FPG sensitive sites were higher in evening time compared to morning time (Student’s t-Test for paired samples, p < 0.05) (Fig. 3B).

### DNA damage repair in morning and evening times

In the Ro 198022 treatment, no significant difference in the repair of SSBs was found between morning (8:00 AM) and evening (8:00 PM) lymphocytes (ANOVA repeated measures two way, p > 0.05) (data not shown). On the contrary, FPG sensitive sites were repaired at faster rates in lymphocytes collected at 8:00 AM than those at 8:00 PM (ANOVA repeated measures two way, p < 0.05) (Fig. 3C). The difference in the rate of repair of FPG sensitive sites between the two time points after 180 min results in about 30% higher residual damage at 8:00 PM than at 8:00 AM (68% vs 39% residue damage, respectively). The *Ogg1* expression gradually decreased in both morning and evening lymphocytes over time after DNA damage induction by Ro 198022 treatment (ANOVA repeated measures two way, p < 0.05) but no difference was found in each point between morning and evening lymphocytes (Fig. 3D).

### Expression of selected genes of BER system in shift workers

A significant difference was found in *Ogg1* gene expression in shift workers compared to daytime workers [15.3 ± 27.5 vs 43.2 ± 23.9 (2^−ΔCT^ × 10^3^)] (Student’s t-Test for independent samples, p < 0.05). No significant differences were observed for mRNA levels of *Apex1* [7.7 ± 2.7 vs 7.1 ± 2.2 (2^−ΔCT^ × 10^3^)] and *Xrcc1* [45.9 ± 30.1 vs 37.9 ± 21.4 (2^−ΔCT^ × 10^3^)].

### *In vitro* expression of clock genes and of selected genes of BER system

**Figure 4:**
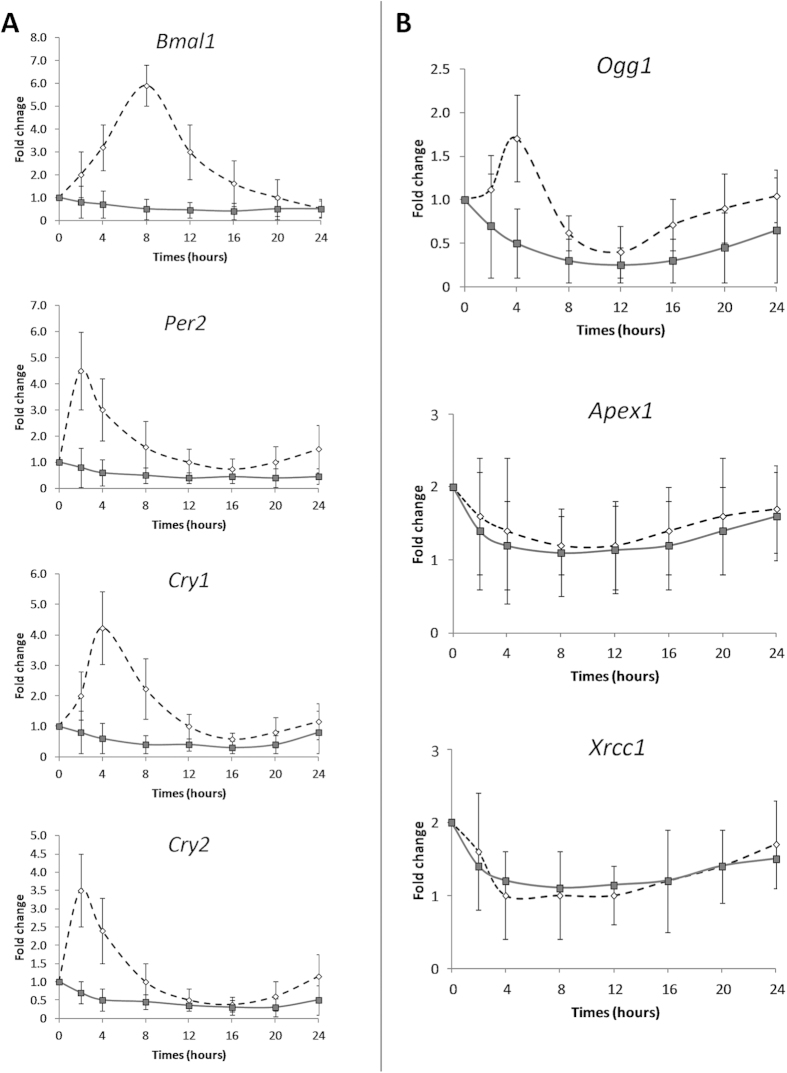
Expression profiles of core clock genes in control (dashed line) HuDe (cell line transfected using a shRNA with a scrambled sequence) and BMAL1-KO (continuous line) HuDe (cell line transfected with shRNA specifically targeting BMAL1) in a 24 h period. Data are expressed as fold change of mRNA levels with respect to time 0 set to 1. At time 0, serum shock was given for 2 h, then cells were kept in starved medium and collected at 2, 4, 8, 12, 16, 20 and 24 hours. A statistically significant circadian rhythm was validated for mRNA levels of clock genes examined ANOVA repeated measures and Cosinor analysis (p < 0.05) in control HuDe but not in BMAL1-KO HuDe.

**Figure 5:**
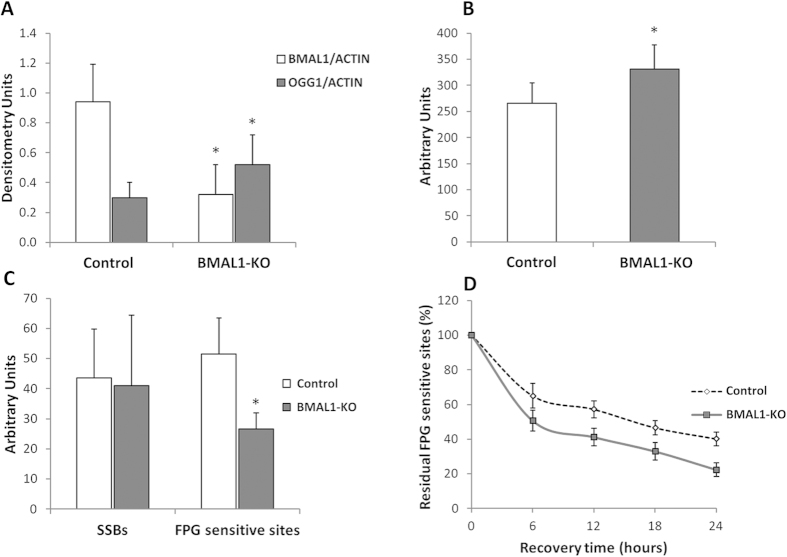
(**A**) Mean (±SD) densitometry of BMAL1 and OGG1 protein in control HuDe (cell line transfected using a shRNA with a scrambled sequence) and BMAL1-KO HuDe (cell line transfected with shRNA specifically targeting BMAL1) * = p < 0.05 BMAL1-KO vs control HuDe. (**B**) Mean (±SD) level of OGG1 activity in control and BMAL1-KO HuDe. * = p < 0.05 BMAL1-KO vs control HuDe. (**C**) Mean (±SD) level of SSBs and FPG sensitive sites in control and BMAL1-KO fibroblasts. * = p < 0.05 BMAL1-KO vs control HuDe. (**D**) The kinetics of repair of FPG sensitive sites at 0, 6, 12, 18 and 24 hours after *ex vivo* oxidative treatment (Ro 198022). The kinetics of DNA repair was calculated as a percentage of residue damage with respect to the damage at 0 minutes. * = p < 0.05 BMAL1-KO vs control HuDe.

The silencing efficiency of the BMAL1 protein was confirmed by Western blot analysis. The densitometric analysis showed a 70% reduction in BMAL1 levels in BMAL1 knockdown (BMAL1-KO) HuDe (human dermal fibroblasts) [cell line transfected with short hairpin RNA (shRNA) specifically targeting BMAL1] compared to control HuDe (cell line transfected using a shRNA with a scrambled sequence). Our results reported a significant variation in mRNA levels of *Bmal1*, *Per2*, *Per3*, *Cry1*, *Cry2*, and *Ogg1* (ANOVA repeated measures, p < 0.05) in control HuDe but not in BMAL1-KO HuDe (Fig. 4). No significant variation was observed for gene expression of *Apex1* and *Xrcc1*. The silencing of BMAL1 protein was associated to an increase of mRNA (1.5 fold), protein (2 fold) and activity of OGG1 (1.4 fold) (Student’s t-Test for independent samples, p < 0.05) leading to a lower levels of FPG sensitive sites. BMAL1-KO HuDe showed a faster rate of 8-oxoG repair resulting in a lower residue damage compared to control HuDe after a 24 h recovery period (20% vs 40%, respectively; two way ANOVA repeated measures, p < 0.05) (Fig. 5).

## Discussion

The genome of mammalian cells is under constant threat of damage by reactive oxygen species that are continuously generated under physiological condition and in response to xenobiotic exposure. We hypothesized that in the context of evolution, daily changes in oxidative stressors may have forced the system involved in DNA damage repair such as the BER pathway to evolve in a circadian fashion to ensure maximum protection during the active phase of the day (morning and afternoon) when there may be a higher probability of oxidative injury.

The goal in undertaking this study was to find out whether 8-oxoG DNA damage repair displayed a circadian rhythm. Firstly, we tested this hypothesis on a group of 15 young healthy subjects. Consistent with previous studies, the concentration of melatonin peaked during night sleep while the level of cortisol peaked in the early morning concurrent with waking^21^,^22^,^23^,^24^,^25^.

However, among our subjects, peak melatonin occurred at 4:00 AM. This is later than the ~2:00 AM reported elsewhere in the literature, and may be a consequence of our light/dark (LD) cycle which used lights out at midnight. Our results are consistent with the results of Wright *et al.*^26^ in which subjects measured in their regular city environment showed melatonin midpoint of 4:00 AM whereas after a week without any electric light (camping) it shifted to 2:00 AM.

Clock genes in lymphocytes showed expression patterns corroborated by the literature^21^,^27^,^28^,^29^,^30^. *Clock* gene exhibited no circadian rhythmicity as previously observed by Bjarnason *et al.*^22^. Taken together, these data suggest that the subjects enrolled in this study were well synchronized along a 24 h time scale.

A daily rhythm for the BER system was inferred primarily by the oscillation of the OGG1 enzyme. The daily variation of OGG1 activity resulted from different levels of mRNA and protein expression. Nevertheless, there was an inter-subject variability in OGG1 activity: some subjects seemed to possess substantial OGG1 rhythmicity in their lymphocytes, while others possessed a less-pronounced OGG1 rhythm. However, no subject lacked transcription of the *Ogg1* gene, and the time of individual peaks and troughs of OGG1 activity appeared to be essentially conserved in the subjects group. It must be stressed that although our results have shown a daily cycle of OGG1 activity consistent with circadian rhythmicity, they do not unequivocally reveal that this activity is endogenous because the subjects were maintained in the laboratory on a LD cycle (i.e., LD 16:8). Similarly, DNA damage repair showed daily variation in lymphocytes taken from these same volunteers. A constant routine protocol will be required for final demonstration of endogenous circadian rhythmicity.

Human OGG1 activity in lymphocytes displayed about 50% variation along a 24 h time period with the lowest value in the evening and the highest early in the morning, near the start of subject’s active span. The physiological downregulation of OGG1 activity in the evening hours resulted in higher endogenous levels of 8-oxoG than those in the morning hours. Two previous studies of daily variation in DNA repair have used mouse brain, and mouse skin^14^,^31^. Interestingly, the findings in each showed peak nucleotide excision repair (NER) activity in the evening, in contrast with our finding evidence of peak BER activity in the morning in humans. Another study^32^ examined antioxidant gene expression and GSH level in mouse liver, and found peak GSH in the morning, whereas mRNA levels of NRF2 pathway peaked in the afternoon.

**Figure 6:**
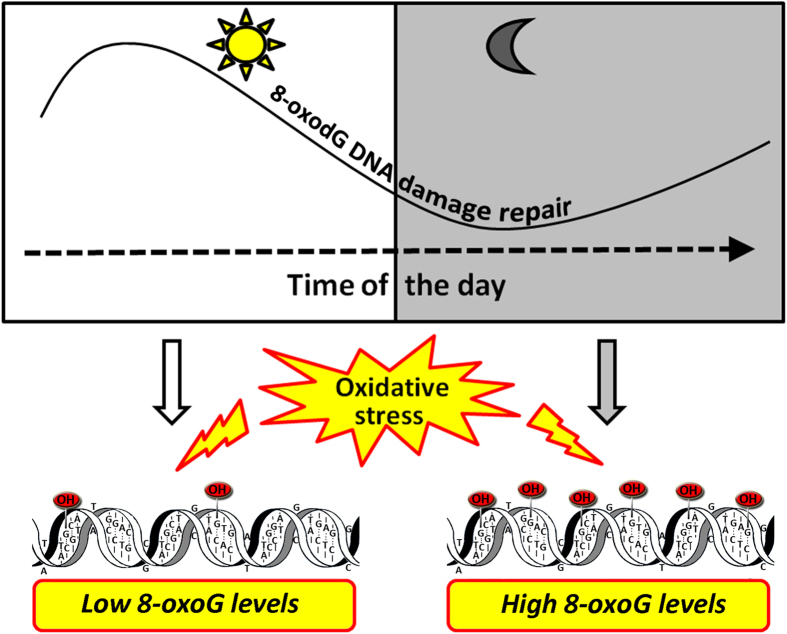
Model for the role of the circadian time in 8-oxoG repair. The higher level of OGG1 activity in the morning hours renders humans less susceptible to accumulate 8-oxoG DNA damage in this time of the day.

Our *ex vivo* results showed that lower OGG1 activity in the evening hours led to a slower repair capacity of oxidative damage than in the morning hours with higher 8-oxoG accumulation. The difference between the rate of repair in the morning and in the evening is significant only for FPG sensitive sites, consistent with the notion that a decrease in OGG1 levels has a more profound effect on the rates of repair of the poorly recognized 8-oxoG than the efficiently recognized SSBs, probably due to the presence of other enzymes (besides OGG1) capable of repairing SSBs. These results are summarized in Fig. 6.

Interestingly, exposure to oxidative stress did not upregulate a transcriptional response of *Ogg1* expression needed to face DNA damage even during evening times when the repair capacity is at its lowest levels. This evidence supports the hypothesis that *Ogg1* expression is not modulated in response to a DNA damage^16^, but it could follow a circadian variation exclusively regulated directly or indirectly by the endogenous biological clock.

Our *in vitro* results showed that *Ogg1* expression followed a circadian rhythmicity in phase with *Per* and *Cry* genes in line with the results obtained *in vivo*. In BMAL1-KO HuDe the loss of circadian rhythmicity of clock gene expression is paralleled by the loss of *Ogg1* expression fluctuation, pointing out the dependence of *Ogg1* modulation on a correct functioning of the molecular circadian clock. It was previously reported that BMAL1 deficiency affects cell survival both from hydrogen peroxide (higher sensitivity) and anti-cancer agents (lower sensitivity)^33^. BMAL1-KO fibroblasts also showed decreased DNA damage induced by DNA-damaging anticancer drugs^34^. Our findings further implicate BMAL1 in the regulation of the BER pathway reflected in an association between the suppression of *BMAL1* expression and an increase of OGG1 activity resulting in a higher rate of 8-oxoG repair in response to oxidative injury. Circadian gene KOs which obliterate circadian rhythmicity in mouse and cell models may be inherently different from the circadian disruption which occurs when an otherwise normal human is exposed to light at night.

Individuals who work at night (such as shift workers) experience exposure to light and have altered sleep/wake cycles resulting in impaired physiological rhythms^11^. However, the connection between internal circadian desynchronization (for example in shift workers) and cancer etiology remains unresolved and controversial^15^. Altered clock gene expression was previously reported in shift workers^35^. This alteration in the mRNA levels of clock genes may be potentially due to an epigenetic impact of shift work^36^. In this study, we found that shift workers experiencing altered circadian clock showed an alteration of *Ogg1* expression. The asynchronous manner of clock gene regulation in shift workers would not be directly responsible for malignant disease, but a lack of protection may allow toxic exposures to affect cells that are in a more vulnerable state.

Our findings support that night workers should avoid any environmental and lifestyle agents potentially capable of generating any DNA damage such as cigarette smoking which was found to affect the oxidized DNA levels in the sperm^37^.

Our study shows some limitations to be taken in consideration. To avoid excessive blood sample collection, we studied selected genes of clock and BER system, focusing on the *Ogg1* gene as limiting enzyme of BER system. As consequence we cannot rule out a circadian variation of other genes involved in BER pathway. However our findings led us to infer the daily rhythm for the BER system primarily by the oscillation of the OGG1 enzyme.

Moreover we cannot know if differences of clock genes and *Ogg1* gene expression found between shift and daytime workers in a single time point were due to variations in amplitude or phase shift of circadian gene expression, or if they persist in other time points in the 24 h period. The limitation of the single time point of sampling is a common issue in studies aiming to investigate an expression of genes with a circadian variation.

In conclusion, for the first time, the findings of this study provide evidence of a circadian regulation of the BER system mainly as a result of the oscillation of the OGG1 enzyme and suggest that people with a desynchronization of circadian rhythms (i.e. shift workers) may experience an alteration of oxidative DNA damage repair. This may also occur to some extent in all persons in the modern world who use electricity to light the night.

## Methods

The study was approached in three ways. First, we examined circadian variation in 8-oxoG repair in lymphocytes of 15 healthy volunteers over a 24 hour period. Second, from a previous study, we measured the expression of *Ogg1* in lymphocytes of 60 rotating shift workers and 54 daytime workers from a blood sample taken in the morning for all workers after a day off. Finally, we then used human fibroblasts *in vitro* to test the influence of BMAL1 in circadian cycling of *Ogg1* expression and in repair of 8-oxoG induced by oxidative damage to DNA.

### Participants and sampling

We enrolled 15 healthy subjects (8 males and 7 females) aged 27–39 [mean ± standard deviation (SD): 32.4 ± 4.3 years]. All subjects filled out a questionnaire which also included their informed consent. The study was carried out according to the Declaration of Helsinki, and the samples were processed under the approval of the written consent statement (Prot. n° 737) by the Ethical Committee of Catania, Italy.

All subjects must have had regular sleep/wake patterns and no history of severe physical diseases; health status was controlled with a physical examination. No subjects had traveled across time zones or had been on medication in the past two months. For 7 days prior to being admitted to laboratory, subjects maintained their daily routines and slept for 8 hours at regular times each night in the absence of artificial light at home. All female have to be in the early follicular phase (between the 2^nd^ and 5^th^ day of the menstrual cycle). Subjects entered the laboratory at 8:00 AM and remained there for a 24-hour period. Subjects were allowed to move, eat and drink ad libitum from 8:00 AM to 12:00 AM (awake time) and could sleep on a bed in the same room from 12:00 AM to 8:00 AM (sleep time). Light intensity in the laboratory was measured at the eye level by Minolta Chroma Meter CL-100 (Minolta Camera Company, Ltd. of Osaka, Japan). During wake time light intensity was 407.7 ± 112.5 lux (mean ± SD), the light was provided by 4000 K fluorescent lamps (Osram Lumilux, Osram, Munich, Germany) while during sleep time light intensity was 2.6 ± 2.2 lux (mean ± SD) coming from a bulb emitting red (700 K) light (Philips PAR38 IR, Philips Lighting, Eindhoven, The Netherlands). Ambient temperature was maintained at 22 ± 1 °C throughout the study. Blood was collected in an EDTA glass tube every 4 h for a 24 h period (8:00 AM, 12:00 PM, 4:00 PM, 8:00 PM, 12:00 AM, 4:00 AM and 8:00 AM). Immediately following blood sampling, lymphocytes and plasma were collected.

Nocturnal samples (4:00 AM) were obtained under a light intensity of 49.1 ± 8.7 lux provided by a bulb emitting red (700 K) light (Philips PAR38 IR, Philips Lighting, Eindhoven, The Netherlands). The circadian synchronization of each subject was verified by assessing the rhythms of plasma cortisol and melatonin.

Selected genes involved in the BER system (*Ogg1, Apex1, Xrcc1*) were further investigated in the lymphocytes of 60 shift workers and 56 daytime workers enrolled in a previous study^35^. Nurses were evaluated and selected based on the following criteria: fertile age (presence of a menstrual cycle); no current treatment with drugs; a negative history of psychiatric disorders, degenerative or cardiovascular diseases, insomnia, chronic viral infections, tumor or autoimmune diseases, or conditions such as fibromatosis of the uterus and polycystic ovary; no occupational exposure to ionizing radiation or involvement in antiblastic drug preparation; and absence of artificial light when sleeping at home. Shift nurses had to be assigned for at least two years to the current shift schedule involving at least 60 night-shifts/year without schedule breaks in the previous 6 months. Daytime nurses had to have a habitual sleep/wake schedule between approximately 11:00 PM and 6:00 AM with no episode of sleep deprivation for at least 3 weeks prior to the study. The samples from nurses in both shift and daytime groups were taken at the beginning of the morning-shift after a regular night’s sleep on a day off. All participants were in the early follicular phase (between the 2^nd^ and 5^th^ day of the menstrual cycle). Shift workers were employed in a “forward (clockwise) rapidly rotating” type of shift work, and the schedule was as follows: day 1: 7:00 AM – 2:00 PM; day 2: 2:00 PM – 10:00 PM; day 3: 10:00 PM – 7:00 AM; 48 hours of rest; resumption of the cycle. The work schedule of daytime workers was from 7:00 AM to 2:00 PM six days/week. Fasting blood (8 h) sampling was performed at 7:00 AM, at the beginning of the morning shift after a day off. Samples were processed immediately after collection for isolating lymphocytes and stored at −80 °C until gene expression analysis.

### Cortisol and Melatonin assays

Plasma levels of cortisol and melatonin were determined by an immunoassay from DRG International Inc. (Mountainside, NJ, USA) according to the manufacturer’s instructions. All samples were measured in duplicate. Inter-assay precision (CV) of these analyses were all <20%.

### Gene expression analysis

The isolation of total RNA was performed (OriGene, Rockville, MD, USA) according to the manufacturer’s instructions. RNA quality and quantification were evaluated with a Nanodrop 1000 spectrophotometer (Thermo Scientific, Wilmington, USA). cDNA was synthesized according to the High-Capacity cDNA Reverse Transcription Kit protocol (Applied Biosystems, Foster City, USA). The genes investigated were: *Bmal1, Clock, Per1, Per2, Per3, Cry1*, *Cry2, Rev-Erbα* (clock genes) and *Ogg1, Apex1, Xrcc1* (selected genes of the BER system). Gene expression was analyzed by real-time quantitative PCR using the TaqMan Gene Expression Master Mix (Applied Biosystems, Foster City, USA). Specific primer sets were obtained from IDT (Integrated DNA Technologies Inc., Coralville, USA). Glyceraldehyde-3-phosphate dehydrogenase (*Gapdh*) was used as endogenous control. The relative mRNA expression levels were calculated applying the following equation: 2^−∆Ct^, and the fold change value of expression compared to a control was calculated following the ΔΔCt method^38^.

### OGG1 activity analysis

The activity repair of oxidative damage to DNA was assayed on cellular extract (containing DNA repair enzymes) from lymphocytes collected from each participant enrolled in the study and from BMAL1-KO HuDe (cell line transfected with shRNA specifically targeting BMAL1) and control HuDe (cell line transfected using a shRNA with a scrambled sequence) by using a modification of the Comet assay^39^.

The repair capability is measured by the occurrence of breaks production on the specifically damaged DNA substrate. Incision activity of the cell extract was estimated as Arbitrary Units (AU) by subtracting the value without extract protein from the value with extract protein. Each value of AU was calculated as % relative value compared to max value set to 100%.

### Oxidative damage to DNA analysis

Two samples of lymphocytes, collected at 8:00 AM (corresponding to OGG1 peak) and at 8:00 PM (corresponding to OGG1 trough), were analyzed for measuring oxidative damage to DNA and repair. DNA breaks and oxidized purine and pyrimidine bases were measured using the Comet assay described elsewhere^40^. The extent of DNA migration was evaluated by visual scoring by an independent observer. Comets were classified and assigned to five categories (0–4) according to the extent of DNA migration. The number of comets counted on each slide was 100. Each sample was analyzed in duplicate, and the value of oxidative damage was expressed with a range 0–400 Arbitrary Units (AU).

### Repair of oxidative damage to DNA

In order to determine the effect of time of day of oxidative DNA damage on the repair rate of 8-oxoG, repair of oxidative damage to DNA was evaluated by inducing *ex vivo* oxidative damage to DNA in the lymphocytes collected at 8:00 AM and 8:00 PM as well as in control and BMAL1-KO HuDe cells as described elsewhere^41^.

In addition to determining whether the *Ogg1* expression was modulated in response to oxidative stress, *Ogg1* mRNA levels was monitored after Ro 198022 treatment of lymphocytes collected at 8:00 AM and 8:00 PM. At regular intervals (0 h, 1 h, 3 h, 6 h), aliquots of the samples were collected and processed in order to evaluate oxidative damage to DNA and *Ogg1* mRNA levels. The kinetics of DNA repair was calculated as a percentage of residue damage compared to the damage at 0 minutes (T0). Residual damage (%) = [(Tn-T0)/(T0-Tb)] × 100, where Tb and Tn indicate the DNA damage at basal (before Ro198022 treatment) and kinetic time points, respectively.

### *In vitro* cell culture and knockdown of BMAL1

A cell culture of HuDe [purchased from the Istituto Zooprofilattico Sperimentale, Brescia, Italy^42^] were knockdown for BMAL1. HuDe cells were transfected with 1 μg of the PBMAL1 shRNA pRS plasmid, or 1 μg of empty pRS plasmid (scramble shRNA) (OriGene) using the TransIT-LT1 transfection reagent (Madison, WI, USA). For BMAL1 knockdown, shRNA sequences targeting the respective mRNA BMAL1: 5′-CCCTGATGCCTCTTCTCCA-3′ was used. The entrainment of clock gene expression was induced by serum shock stimulation^43^,^44^,^45^. To this end, the cells were starved for 48 h and were later stimulated with a serum-rich medium (50% DMEM and 50% fetal bovine serum) for 2 h, subsequently the cells were cultured in serum-free DMEM and harvested after 0 h, 2 h, 4 h, 8 h, 12 h, 16 h, 20 h and 24 h from start of serum shock for the gene expression analysis.

### Immunoblot analysis

Protein levels were analyzed by immunoblot analysis using anti-BMAL1 IgG (Origene, Rockville, MD, USA), anti-OGG1 (Origene, Rockville, MD, USA) and polyclonal anti-β-actin (Bethyl, Montgomery, TX, USA). Protein bands were visualized using the ECL detection system (Pierce Biotechnology, Rockford, IL, USA). Band intensities were evaluated by ChemiDoc using Quantity One software (BioRad, Hercules, CA, USA).

### Statistical analysis

A total sample size of N = 10 volunteers was calculated a priori to detect significant differences with an effect size of 0.60, a power >0.80, and a α = 0.05 (two-tailed) for all the variables studied. The theoretical sample size was increased by 50% in order to include a satisfactory final number of participants. Variables were expressed as mean ± SD. ANOVA repeated measures was performed to analyze repeated measures at different time points. Mauchly test was performed to verify the sphericity assumption, and LSD test was used as a post-hoc test. Student’s t-Test was used to test differences of independent measures between two groups. Pearson correlation test was applied to analyze relationships between continuous parameters. Cosinor analysis was performed to study the circadian rhythmicity. Statistical significance was set at p < 0.05, and statistical tests were two-sided. We analyzed our data by Statistical Package Social Sciences (version 19) software (SPSS, Chicago, IL, USA) and Circadian software (available on-line at www.circadian.org).

## Additional Information

**How to cite this article**: Manzella, N. *et al.* Circadian Modulation of 8-Oxoguanine DNA Damage Repair. *Sci. Rep.* **5**, 13752; doi: 10.1038/srep13752 (2015).
